# Dichoptic Visual Search at Varied Fellow Eye Contrasts and Visual Function Deficits in Amblyopia

**DOI:** 10.1167/iovs.66.9.39

**Published:** 2025-07-14

**Authors:** Shi Shi, Ibrahim M. Quagraine, Gokce Busra Cakir, Jordan Murray, Aasef G. Shaikh, Fatema F. Ghasia

**Affiliations:** 1 Case Western Reserve University School of Medicine, Cleveland, Ohio, United States; 2 Department of Biomedical Engineering, Case Western Reserve University, Cleveland, Ohio, United States; 3 Cole Eye Institute, Cleveland Clinic, Cleveland, Ohio, United States; 4 Daroff-Dell'Osso Ocular Motility Laboratory, Louis Stokes Cleveland VA Medical Center, Cleveland, Ohio, United States

**Keywords:** amblyopia, visual search, interocular suppression, dichoptic treatments

## Abstract

**Purpose:**

This study uses dichoptic visual search and psychophysical visual function data to understand factors that impact amblyopic eyes’ ability to perceive the stimulus while the fellow eye (FE) contrast is reduced under dichoptic viewing in children and adults.

**Methods:**

Twenty-three amblyopic (anisometropic = 11, strabismic/mixed = 11) and 14 control participants performed visual search and spot-the-difference tasks during monocular and dichoptic viewing with amblyopic eye (AE) contrast held constant at 100% while FE contrasts varied at 100%,50%,25% and 10%. Accuracies and reaction times were analyzed and correlated with visual acuity deficit of amblyopic eye, stereoacuity and suppression.

**Results:**

Amblyopic participants showed reduced accuracies and longer reaction times in visual search and spot-the-difference tasks. Reducing the FE contrast improved their ability to identify the targets and differences with greater improvement observed for anisometropic than strabismic subjects. However, participants with higher suppression, visual acuity deficits in the AE, and stereoacuity deficits faced greater difficulties, with reduced accuracies and reaction time, especially at high FE contrasts. For tasks requiring comparison between AE and FE regions to spot-the-differences, deficits in AE visual acuity, suppression and stereoacuity were linked to reduced accuracy and increased reaction times, even at 10% FE contrasts.

**Conclusions:**

The study highlights the challenges amblyopic participants face in dichoptic tasks, particularly at high FE contrasts. Dichoptic contrast modulation holds promise in enhancing the perception of the AE with responses varying by visual function deficits. It also highlights that quantification of perception of the AE in dichoptic environment with tools such as visual search may allow tailoring of passive video-based dichoptic therapies.

Amblyopia, affecting 3% to 5% of the population, is a neurodevelopmental disorder stemming from mismatched visual input between the eyes during early childhood. This mismatch can result from conditions like strabismus, anisometropia, or visual deprivation. Amblyopia is multifaceted, characterized by reduced visual acuity in the amblyopic (weaker) eye, diminished depth perception, and inter-ocular suppression.^1^^–^^3^ Psychophysical and oculomotor studies have demonstrated that amblyopia affects not only the amblyopic eye (AE) but also, to a lesser extent, the fellow eye (FE). Deficits in the FE include reduced contrast sensitivity and visual acuity,^2^^,^^4^ impaired motion perception, and slower reading speeds.^1^^,^^5^ Conventional treatments have focused on improving the monocular functions of the amblyopic eye through methods such as patching or pharmacological penalization of the fellow eye. However, these approaches often carry limitations, such as psychosocial burdens, treatment noncompliance, and a risk of regression post-treatment.^6^^–^^12^ More importantly, binocular visual function abnormalities frequently persist despite improvements in monocular visual acuity.^8^^–^^10^ Recent research has increasingly recognized inter-ocular suppression as a key factor in the pathogenesis of amblyopia. Visual signals from the FE have been found to actively inhibit those from the AE at the cortical level, impeding binocular combination. Stronger suppression from the FE correlates with greater visual deficits in the AE, including reduced acuity, particularly at higher contrast and spatial frequencies.^13^^–^^15^ In light of these findings, novel binocular treatment approaches have emerged that specifically target inter-ocular suppression and address the binocular nature of amblyopia.

Dichoptic amblyopia therapy, which involves presenting visual stimuli simultaneously and independently to each eye, aims to overcome suppression and promote cortical integration of binocular visual input. This approach uses contrast rebalancing by reducing FE contrast while maintaining AE contrast at 100%, counteracting suppression and facilitating binocular combination. Several trials conducted in adults demonstrated improvement in interocular suppression, increased binocular fusion and stereoacuity in subjects treated with dichoptic therapies.^16^^–^^19^ Although the premise of the dichoptic treatments is a greater reduction in suppression with better stereoacuity than patching, the studies in pediatric patients with anisometropia and small-angle strabismus have produced mixed results.^15^^,^^20^^,^^21^ Poor compliance and high participant dropout rates are potential reasons for the underwhelming results of binocular treatments in childhood amblyopia. To improve compliance in younger children, the dichoptic viewing paradigm has been applied to passively watching videos. Additionally, dichoptic masking—where complementary parts of images are presented to each eye separately—further enhances this effect.^22^^–^^26^ Although passive dichoptic video-based therapies have shown improved compliance, they do not verify whether amblyopic subjects accurately perceive the dichoptic stimuli. Investigating visual performance under dichoptic viewing conditions can yield valuable insights that complement dichoptic training approaches. Previous studies from our lab and others have demonstrated that amblyopia impairs visual search performance, with slower visual processing times observed under both binocular and fellow eye viewing conditions.^27^^–^^30^ However, no studies have yet evaluated how dichoptic manipulation of fellow eye stimuli affects visual search and the amblyopic eye's ability to perceive stimuli in children.

The purpose of our study aims to use dichoptic visual search and psychophysical visual function data to identify factors that affect AE's perception of dichoptically presented stimuli when FE contrast is reduced, and dichoptic masking is applied. We hypothesize that visual search performance will be more significantly impaired in the absence of fellow eye contrast manipulation compared to when viewing with the amblyopic eye alone. This anticipated impairment is attributed to the suppression exerted by the fellow eye on the amblyopic eye. Additionally, we also hypothesize that individuals with greater visual acuity deficits will exhibit higher levels of suppression and stereoacuity deficits, resulting in greater difficulty perceiving dichoptic stimuli even at reduced FE contrast levels.

## Methods

The experiment protocols complied with the tenets of the Declaration of Helsinki and were approved by the Cleveland Clinic Institutional Review Board. Informed consent was obtained from the study participants and parents or legal guardians on behalf of minors/children. We recruited 37 subjects (controls = 14 and amblyopia = 23). All participants underwent a comprehensive eye examination, and clinical parameters were collected during the exam (Table 1). They wore optical corrections based on their cycloplegic refraction, with hyperopic adjustments made symmetrically for both eyes as clinically necessary. The type and severity of amblyopia was classified per the Pediatric Eye Disease Investigator Group^31^ as treated/recovered (*n* = 5), mild (*n* = 5), and moderate/severe (*n* = 13). Subjects with mild amblyopia and those with treated amblyopia are analyzed collectively as mild/treated. Amblyopia was categorized by etiology as anisometropic (*n* = 11) and strabismic/mixed (*n* = 12). Subjects with amblyopia and those with treated amblyopia are collectively described as amblyopic participants.

**Table 1.** Demographic and Visual Function Data of Amblyopic Participants

Subject Number	Sex	Age	Type	Visual Acuity (AE) logMAR	Visual Acuity (FE) logMAR	Severity of Amblyopia	Refraction (OD)	Refraction (OS)	Strabismus Distance (∆)	Stereopsis (Log Arc Sec)	Dichoptic Motion Coherence (Log Cum AUC
1	M	11	Aniso	0.309	0.004	Moderate	2.50 + 1.75 × 103	4.50 + 1.25 × 073	Ortho	2.00	3.00
2	F	12	Aniso	0.426	−0.056	Moderate	1.75 + 0.50 × 110	5.50 + 2.00 × 080	Ortho	2.30	3.23
3	F	32	Strab	0.405	0.043	Moderate	7.00 + 0.50 × 147	7.00 + 0.50 × 057	RHT8, RET2	3.54	2.87
4	M	9	Mixed	0.077	−0.023	Treated	2.00 + 1.50 × 105	0.50 + 0.75 × 080	RX(T)14 LH(T)6	2.00	3.03
5	F	9	Aniso	0.331	0.072	Moderate	Plano + 4.00 × 095	0.25 + 2.25 × 080	Ortho	2.00	3.13
6	F	22	Strab	0.013	−0.088	Treated	7.25	6.50 + 0.75 × 030	Flick LHT	3.85	2.23
7	F	13	Mixed	0.237	−0.036	Mild	−2.25 + 0.75 × 100	−3.50 + 1.50 × 080	2XT Flick RHT	2.00	3.13
8	F	11	Aniso	0.231	0.00	Mild	−0.75 + 1.50 × 082	−2.75 + 6.25 × 088	Ortho	2.15	1.81
9	F	11	Aniso	0.286	0.160	Mild	5.00 + 1.50 × 096	6.00 + 1.25 × 088	Ortho	2.00	2.36
10	F	13	Mixed	0.488	0.012	Moderate	2.25 + 1.50 × 115	0.50 + 0.75 × 080	X(T) flick	2.00	3.13
11	M	13	Mixed	0.564	−0.097	Moderate	1.75 + 2.50 × 110	0.25 + 0.75 × 085	RET 2, Right hypo 8	3.85	2.49
12	F	12	Mixed	0.176	−0.040	Mild	5.00 + 1.75 × 075	4.00 + 0.75 × 100	RET 4	2.00	2.02
13	M	17	Strab	0.331	−0.023	Moderate	1.25 + 4.50 × 095	1.00 + 1.75 × 090	LE(T) 6–8	3.85	3.21
14	M	10	Aniso	0.192	0.006	Mild	0.75 + 0.50 × 095	2.50 + 1.75 × 075	Ortho	2.00	2.41
15	F	11	Aniso	0.359	−0.108	Moderate	1.25 + 2.00 × 095	0.50 + 0.25 × 085	Ortho	2.90	3.08
16	F	30	Mixed	−0.093	−0.003	Treated	2.25 + 0.75 × 136	−0.25 + 1.50 × 079	2 E(T), 12 DVD	3.85	1.69
17	F	7	Aniso	0.773	0.057	Severe	2.50 + 0.25 × 090	4.50 + 0.50 × 085	Ortho	2.90	3.12
18	M	7	Aniso	0.161	0.024	Mild	1.75	4.5 + 0.50 × 126	Ortho	2.00	1.88
19	M	9	Strab	0.313	0.076	Moderate	7.5 + 0.5 × 71	7.00 + 1.00 × 130	Flick E	2.00	2.45
20	M	9	Mixed	0.449	−0.023	Moderate	1.25 + 3.75 × 095	0.50 + 2.00 × 085	RET 4–6	2.30	3.28
21	M	7	Aniso	0.308	−0.005	Moderate	4.75 + 1.00 × 80	2.50 + 1.00 × 80	Ortho	2.00	2.85
22	M	14	Aniso	0.111	0.089	Treated	3.75 + 1.50 × 65	1.00 + 2.00 × 100	Ortho	2.00	3.10
23	F	30	Strab	0.51	0.12	Moderate	Plano + 0.75 × 11	0.25	14–16 ET	3.85	3.90

Aniso, anisometropic amblyopia based on meeting at least one of the following criteria: ≥ 0.50 D difference between both eyes in spherical equivalent or ≥ 1.50 D difference between both eyes in astigmatism at any meridian; Strab, strabismic amblyopia based on meeting at least one of the following and criteria is not met for mixed amblyopia (see below): (a) heterotropia at distance (with or without spectacles), (b) history of strabismus surgery, and (c) history of strabismus that has resolved with glasses and/or surgery; Mixed, mixed amblyopia based on meeting both of the following criteria: (a) criteria for strabismus (see above), (b) ≥ 1.00 D difference between both eyes in spherical equivalent or ≥ 1.50 D difference between eyes in astigmatism in any meridian. Severity of amblyopia is classified into two groups, Treated (for participants with treated amblyopia) and Mild, Moderate, and Severe as per PEDIG studies; Ortho, Orthotropia; (), intermittent deviation; XT, exotropia; ET, esotropia; HT, hypertropia; HypoT, hypotropia (preceded by L – left and R – right); FE, fellow eye; AE, amblyopic eye, log cum; AUC, log of cumulative area under the curve (AUC) value, ∆ = prism diopters.

Absence of stereopsis (nil) is indicated by a logStereopsis value of 3.85 (represents 7000 arcsec).

### Visual Function Measurements

#### Interocular Suppression

Dichoptic motion coherence testing was used to assess suppression using an FPR LCD Display and polarized glasses.^32^^,^^33^ Signal dots at 100% contrast were presented to the amblyopic eye, whereas noise dots at varying contrasts (100%, 90%, 75%, 62.5%, 56.7%) were shown to the fellow eye. The dichoptic motion coherence threshold at each contrast was determined by the proportion of signal to noise dots required for accurate motion direction identification. Suppression was quantified by fitting a third-order polynomial to the log of the number of signal dots required at each noise contrast level and calculating the area under the curve.

#### Visual Acuity

Right and left eye visual acuity was assessed using ETDRS optotypes with crowding bars on a 32-inch LCD monitor (1920 × 1080 resolution, 120 Hz, 111 cd/m² brightness) at 3.1 meters in a dark room.^4^^,^^13^^,^^14^ This method adjusts the optotype size, measured in arcmin, based on the subject's responses using a two-down/one-up staircase approach. The step size decreases by 50% before the first reversal, then changes by 25% increments and 12.5% decrements, for a total of six reversals. The calculations in arcmin were then converted to logMAR to quantify acuity.

#### Stereoacuity

Stereoacuity was measured in log arcsecs using the Titmus Stereoacuity Test, as previously described.^8^^,^^13^ Participants with absent stereoacuity were assigned a value of 3.85 log arcsecs.

### Visual Tasks

Subjects were presented images on a 32 inches 3D LCD display (1920 × 1080 pixels) with a brightness of 111 cd/m^2^ at a viewing distance of 84 cm. Test stimuli were generated using Psykinematix (Kybervision) software. To deliver different images to the left and the right eye (dichoptic images), inter-leaved polarization was used- every even line was visible to one eye and every odd line was visible to the other eye owing to opposite polarization.

#### Visual Search Task

**Figure 1.**
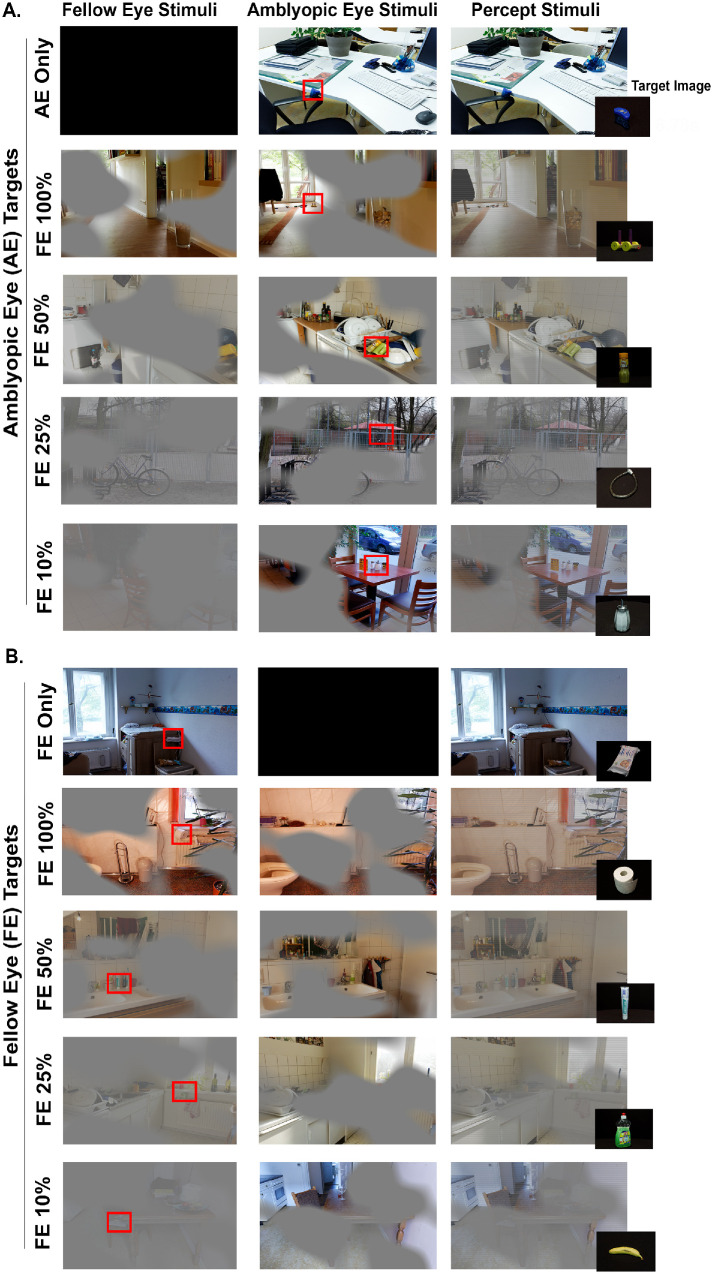
Experimental paradigm for visual search task where the target is within AE region (AE targets; **A**), within FE region (FE targets; **B**). The *left column* depicts FE stimuli, the *middle column* is AE stimuli, and the *last column* is percept of the stimuli. The *red boxes* indicate target of interest.

**S**ubjects were first shown an image of object of interest (Fig. 1) and given 10 seconds to locate that object within a presented image. Once identified, subjects clicked on the object immediately with a mouse. In monocular viewing, the target was only visible to AE or FE with the opposite eye viewing a black screen. Eight images were viewed under monocular viewing, four of which were visible to AE only (Fig. 1A) and four to FE only (Fig. 1B). In dichoptic viewing, masks of irregular shapes were created using a specialized MATLAB program and were applied to the AE (or right eye for the controls) and the inverse to the FE (or left eye for the controls). The shapes had Gaussian edges, causing overlapping areas to be viewed by both eyes at different contrast (Transition Region). It was necessary to piece the stimuli presented to the fellow eye (FE Region) and amblyopic eye (AE Region) together to appreciate the entire image as shown in Figure 1 and as reported previously.^25^^,^^34^^,^^35^ To examine the effect of reducing suppression, four levels of FE contrasts were studied: 100%, 50%, 25%, and 10%. Each FE contrasts contrast level included six images: three with target of interest located within the portion of images visible to the AE (AE Targets) and three within the portion of images visible to the FE (FE Targets). A total of 32 images were used for the visual search task. The sequence of image presentation was random with regards to contrast levels and monocular versus dichoptic viewing.

#### Spot-the-Difference Task

**Figure 2.**
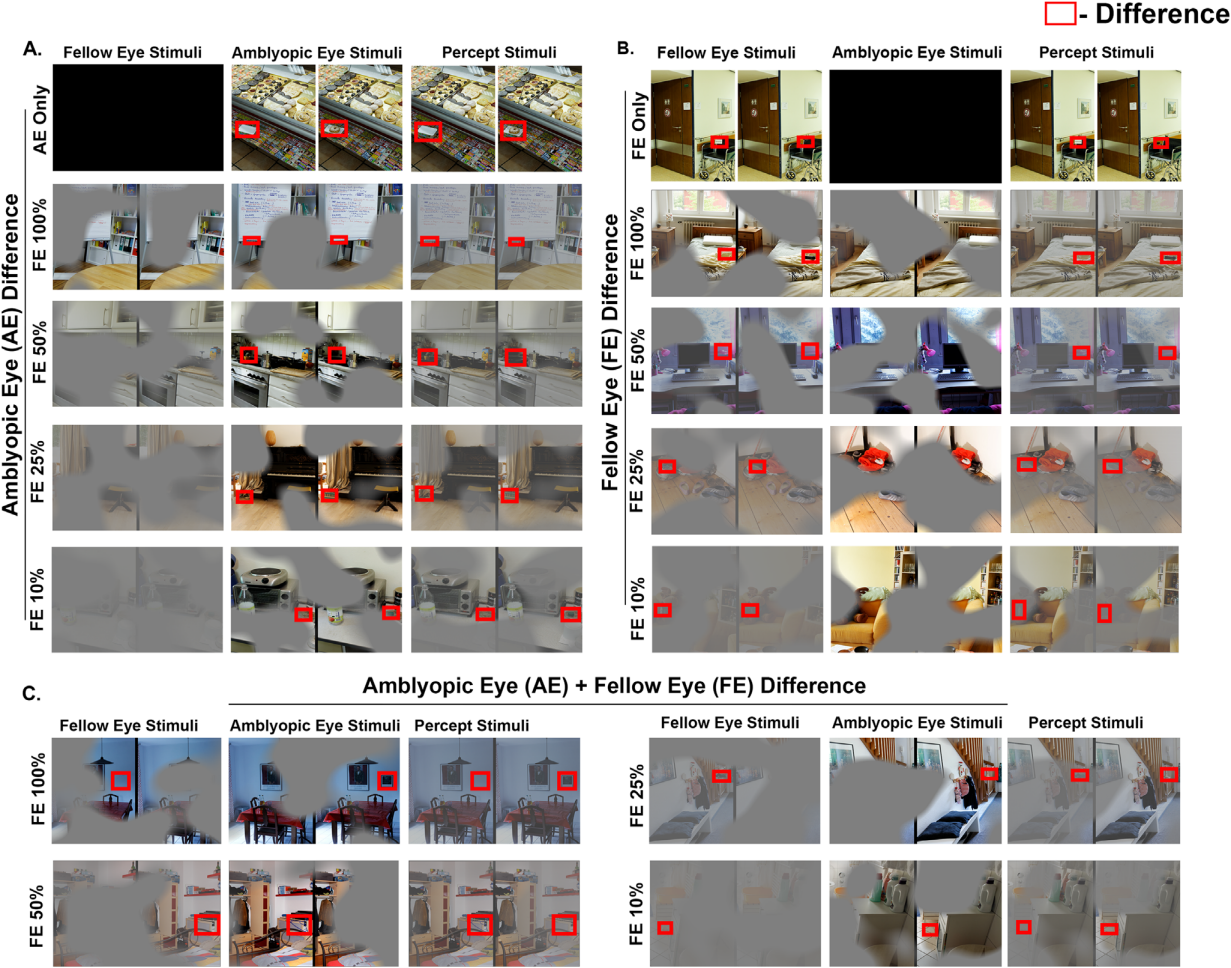
Experimental paradigm for spot the difference task where the difference is within AE region (AE difference; **A)**, within FE region (FE difference; **B**), and where the difference is within FE and AE regions (AE + FE difference; **C)**. The *left column* depicts FE stimuli, the *middle column* is AE stimuli, and the *last column* is percept of the stimuli. The red boxes indicate differences between the two images.

Participants were presented with two nearly identical images side-by-side, with a single difference between them (Fig. 2). They were instructed to identify and click on the difference as soon as they found it, all within a 20-second timeframe. Viewing conditions matched those described for the Visual Search Task. Eight images were viewed under monocular viewing, four of which were visible to amblyopic eye only (AE only) and four to fellow eye only (FE only). Six images per level of FE contrast (100%, 50%, 25%, 10%) were used with three differences within the portion of image visible to the AE (AE Difference) and three differences within the portion of image visible to the FE (FE Difference) (Figs. 2A, 2B). In addition, there are 12 images, three for each level of FE contrast where, subjects need to compare portions of the images visible to the AE and FE to identify the difference between the right and left image called as FE + AE Difference (Fig. 2C). In total, 44 images were used.

### Behavioral Parameters

We analyzed parameters including reaction time and accuracy, defined as the percentage of correctly identified visual targets. Target identification accuracy was recorded live by the experimenter and subsequently verified using mouse click coordinates recorded in MATLAB. Correct clicks fell within an area of interest, defined as a rectangle extending 0.75° beyond the target's edges. Reaction times were defined as the elapsed time from image onset to mouse click. For missed identifications, maximum allowed times (10 seconds for Visual Search; 20 seconds for Spot the Difference) were used for reaction time analyses.

### Statistical Methods

Statistical analysis was conducted using GraphPad Prism. Age between control and amblyopic groups were compared using an unpaired *t*-test. Two-way mixed model ANOVA was used to examine behavioral performance in amblyopia versus control as well as across controls, anisometropic versus strabismic participants, with post hoc Tukey's multiple comparison tests. We conducted Spearman correlation analysis to assess how levels of suppression, stereoacuity and visual acuity of the amblyopic eye correlate with the accuracies and reaction times for each experimental condition. Visual acuity, stereoacuity and suppression levels were compared across ansiometropic versus strabismic participants using an unpaired *t*-test.

## Results

There were no age differences (years) between the control (10.79 ±6.71, age range 8–25 years) and the amblyopia (14.18 ± 7.46, age range 7–30 years, *P* = 0.12) groups.

### Behavioral Performance During Dichoptic Visual Search and Spot the Difference Tasks as a Function of Type of Amblyopia

**Figure 3.**
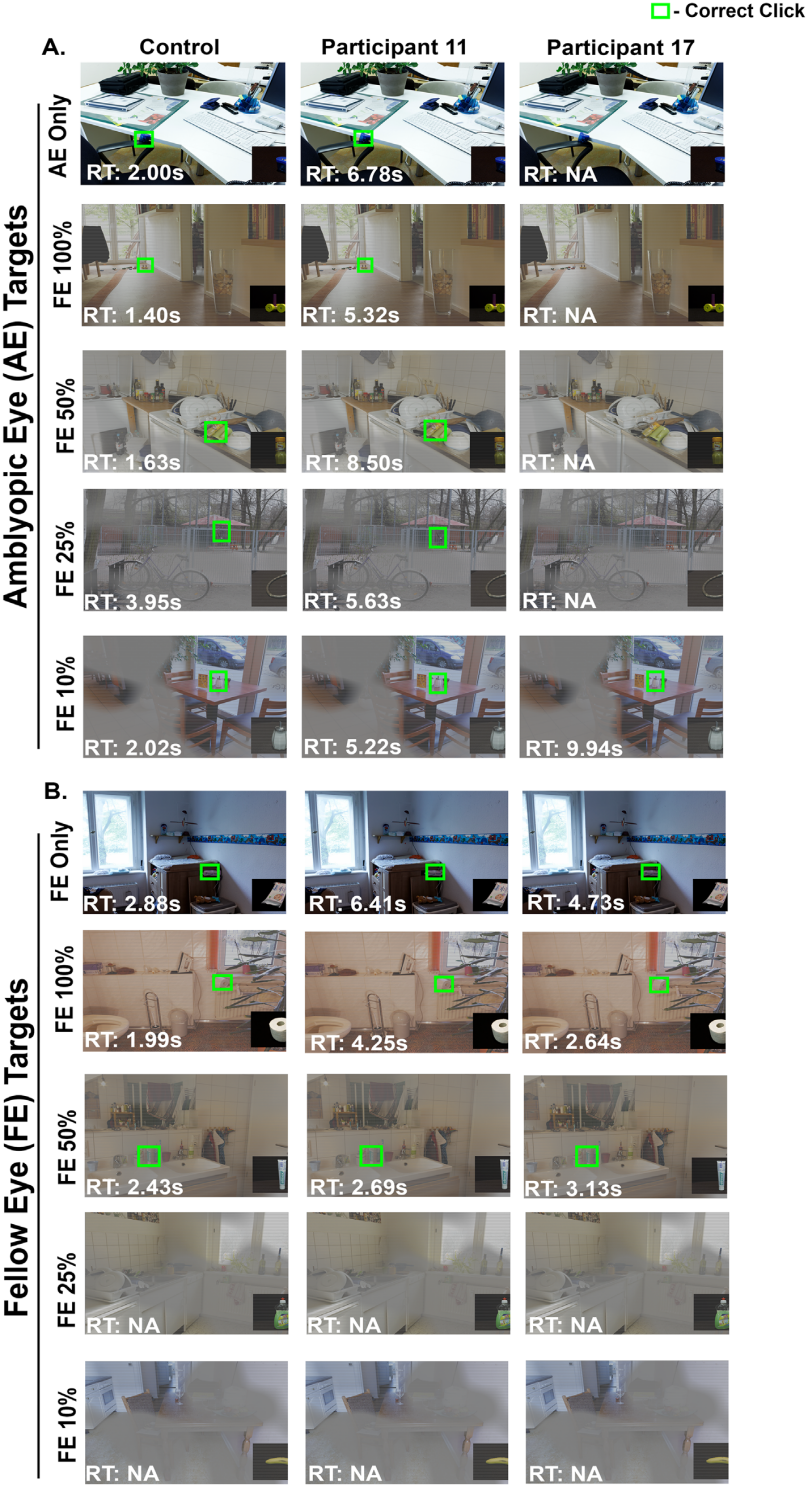
Visual search task performance from a control subject and 2 amblyopic participants for AE targets (**A**) and FE targets (**B**) under monocular (AE only and FE only, respectively) and dichoptic viewing conditions at varied FE contrasts. *Green boxes* indicate accurate target identification with reaction times indicated in the *bottom left corner* of each image. Absence of *green boxes* indicates failure to identify the target.

Figure 3 compares the behavioral performance (accuracies and reaction times) of representative subjects from each group, including a control participant, a strabismic participant with lesser AE visual acuity deficit and suppression (Participant 11, AE Visual Acuity logMAR = 0.56, Suppression = 2.49 Log cumAUC) and an anisometropic participant with greater AE visual acuity and suppression (Participant 17, AE Visual Acuity logMAR = 0.77, Suppression = 3.12 Log cumAUC) . The top five rows depict the percept of the stimuli for trials where the targets are visible only to the amblyopic eye (AE targets), whereas the bottom five rows depict trials where the targets are visible only to the fellow eye (FE targets). The control subject was able to accurately identify all AE targets (green boxes). On the other hand, both amblyopic subjects had difficulties with AE targets. Participant 11, with less visual acuity deficit and suppression, identified AE targets in AE only, as well as when the FE contrast was varied from 100%, 50%, 25%, and 10%. However, the reaction times were increased compared to the control subject. On the other hand, Participant 17, with greater visual acuity deficit, and suppression level, was not able to identify the AE target in AE only and at 100% 50%, and 25% FE contrasts but successfully identified visual target when FE contrast is lowered to 10%. When evaluating the FE target trials, the amblyopic subjects were able to identify the object of interest with longer reaction times; however, lowering FE contrast to 25% and 10% made the stimuli difficult to visualize for all subjects.

**Figure 4.**
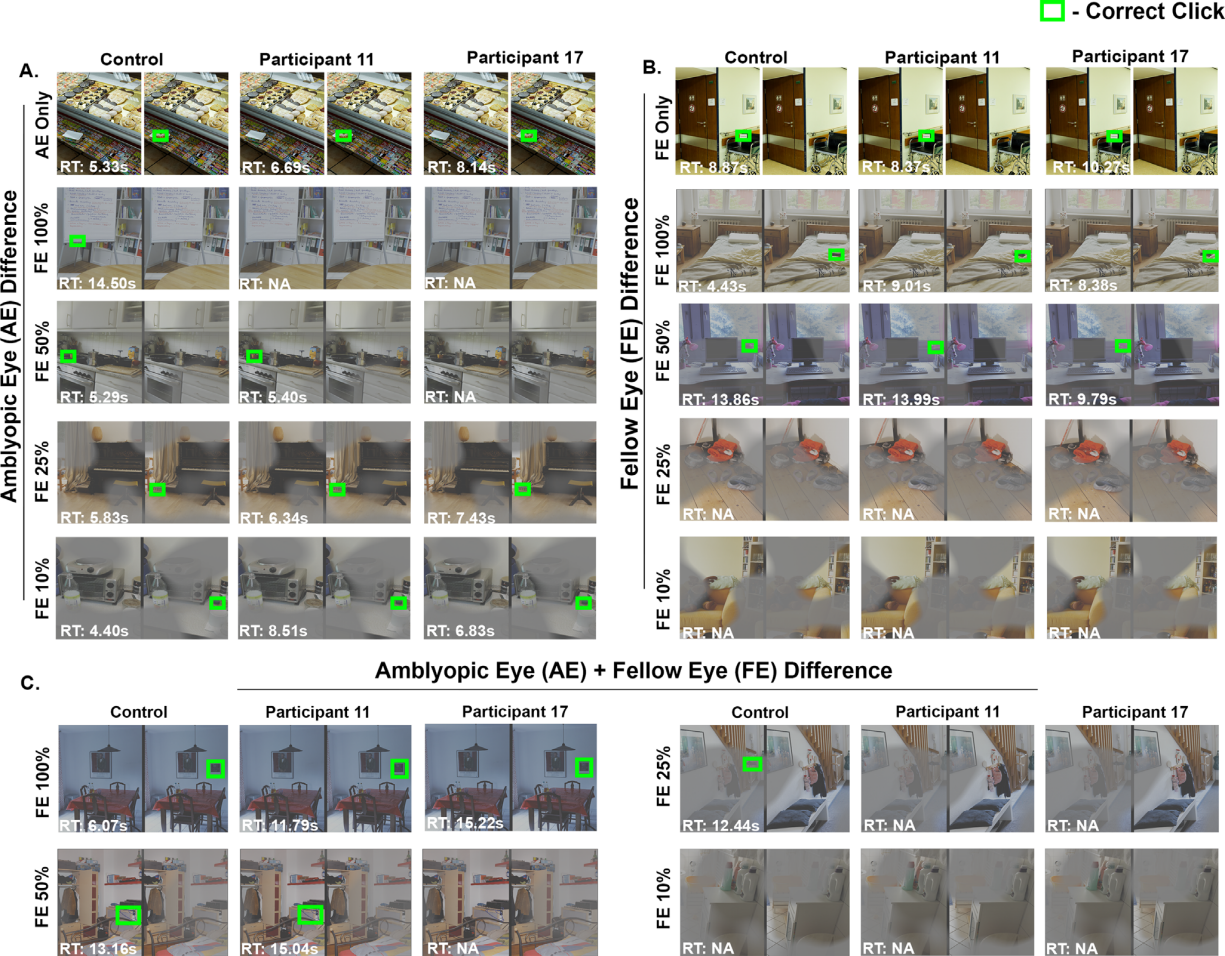
Spot-the-difference task performance from a control subject and two amblyopic participants for AE targets (**A**), FE targets (**B**), under monocular (AE only and FE only, respectively) and dichoptic viewing conditions at varied FE contrasts and AE + FE difference (**C**). *Green boxes* indicate accurate target identification with reaction times indicated in the *bottom left corner* of each image. Absence of *green boxes* indicates failure to identify the target.

Figure 4 shows the percept of the stimuli of the spot-the-difference task, where the complementary masks were placed such that the differences were visible to AE only (AE difference) (Fig. 4A), when the differences were visible to the FE only (FE difference) (Fig. 4B), and when differences are split between AE and FE stimuli (AE+FE difference) (Fig. 4C). Both Participants 11 and 17 successfully spotted the difference in AE only condition but required longer reaction time than control. Neither amblyopic participant was able to spot the difference at 100% FE contrast. As FE contrast was reduced, Participant 11, with less suppression, could spot the difference at 50% FE contrast, whereas Participant 17, with greater suppression, needed a reduction to 25% FE contrast. For FE difference, all three subjects could identify the target in FE only, FE 100%, and FE 50%, but at FE 25% and FE 10%, the stimuli were difficult to visualize for all subjects. Figure 4C depicts percept of the stimuli, where the masks were placed such that the spot the difference images required the participants to perceive the stimuli being presented to both the AE and FE (i.e., AE + FE difference). In the example shown, both amblyopic participants were able to identify the AE + FE differences at 100% FE contrast but exhibited significantly longer reaction times compared to control (Fig. 4C). The control subject successfully detected AE + FE differences at both 50% and 25% FE contrasts. Participant 11 could detect the differences at 50% FE contrast, whereas participant 17 failed to detect the differences at both 50% and 25% FE contrasts. These findings suggest that reducing FE contrast to 25% and 10% is less effective in facilitating the integration of information from both eyes.

**Figure 5.**
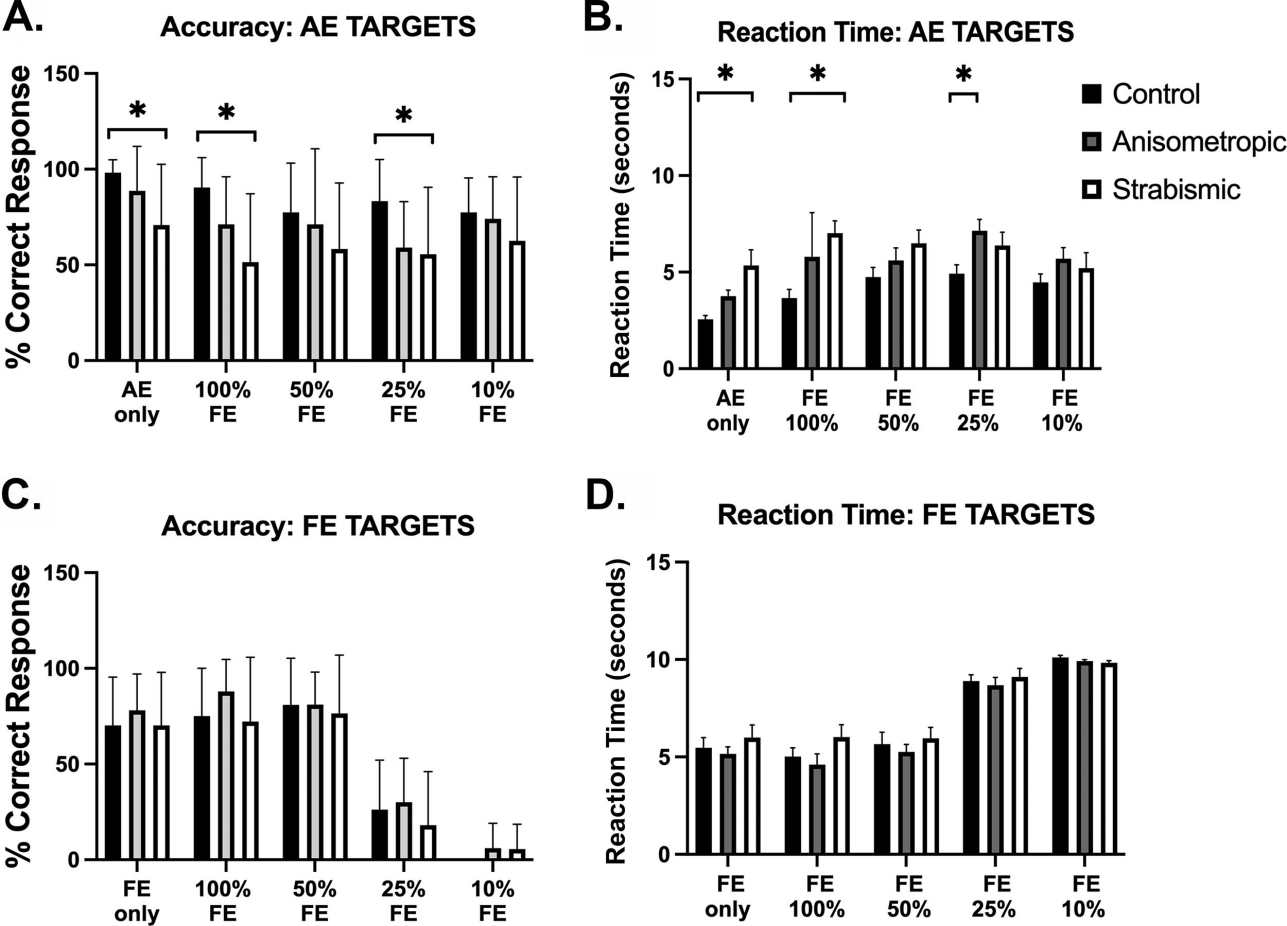
Accuracies and reaction times of controls, anisometropic and strabismic/mixed amblyopic participants in visual search tasks for AE targets (**A** and **B**) and FE targets (**C** and **D**). *Asterisk* indicates statistically significant difference between groups evaluated at *P* < 0.05 for post hoc multiple pairwise comparisons with Tukey correction.

Figure 5 summarizes the accuracies and reaction times of visual search when the target of interest is within the amblyopic eye region (i.e., AE targets; Figs. 5A, 5B) and fellow eye region (i.e., FE targets; Figs. 5C, 5D) in controls, anisometropic amblyopia and strabismic amblyopia participants. In the visual search tasks, for AE target trials, a significant main effect was found for accuracies across the different groups, *F*(2, 170) = 14.48, *P* < 0.0001. Significant differences were also observed within subjects for different viewing conditions for AE targets, *F*(4, 170) = 2.97, *P* = 0.02. No interactions were observed between the amblyopic and control groups and viewing conditions, *F*(8, 170) = 0.59, *P* = 0.78. Post-hoc comparisons revealed that participants with strabismic amblyopia had significantly lower accuracy in identifying AE targets in AE only condition (*P* = 0.02), in 100% FE contrast trials (*P* = 0.0009) and 25% FE contrast trials (*P* = 0.02) compared to controls.

For AE targets, increased reaction times were observed in amblyopia with a significant main effect between groups *F*(2, 160) = 19.34, *P* < 0.0001, as well as within viewing conditions *F*(4, 160) = 5.59, *P* = 0.0003, with no interaction between groups and viewing conditions *F*(8,160) = 1.28, *P* = 0.25. Post-hoc comparisons revealed differences between controls and strabismic amblyopia groups for AE only (*P* = 0.0018) and 100% FE (*P* = 0.0001), as well as increased reaction times were observed between controls and anisometropic amblyopia participants at 25% FE contrast (*P* = 0.01). Increased reaction times were observed at lower FE contrasts with significant differences between AE only and FE 50% (*P* = 0.03), as well as AE only and FE 25% (*P* = 0.01) in control group. Significant differences were also seen between AE only and FE 25% (*P* = 0.007) for anisometropic amblyopia participants.

For FE target trials (Figs. 5C, 5D), no significant main effect of groups on accuracy *F*(2, 170) = 1.90, *P* = 0.15, or reaction time, *F*(2, 160) = 1.18, *P* = 0.39, was observed. Significant effects of viewing conditions was observed for accuracy *F* (4, 170) = 84.69, *P* < 0.00001 and reaction times *F*(4, 170) = 62.20, *P* < 0.00001. Post-hoc analysis revealed significant multiple pairwise comparisons between the various contrast levels for control, anisometropic and strabismic groups, showing that at lower FE contrasts (25% and 10%), participants had reduced accuracy and increased reaction times in both tasks, as expected.

**Figure 6.**
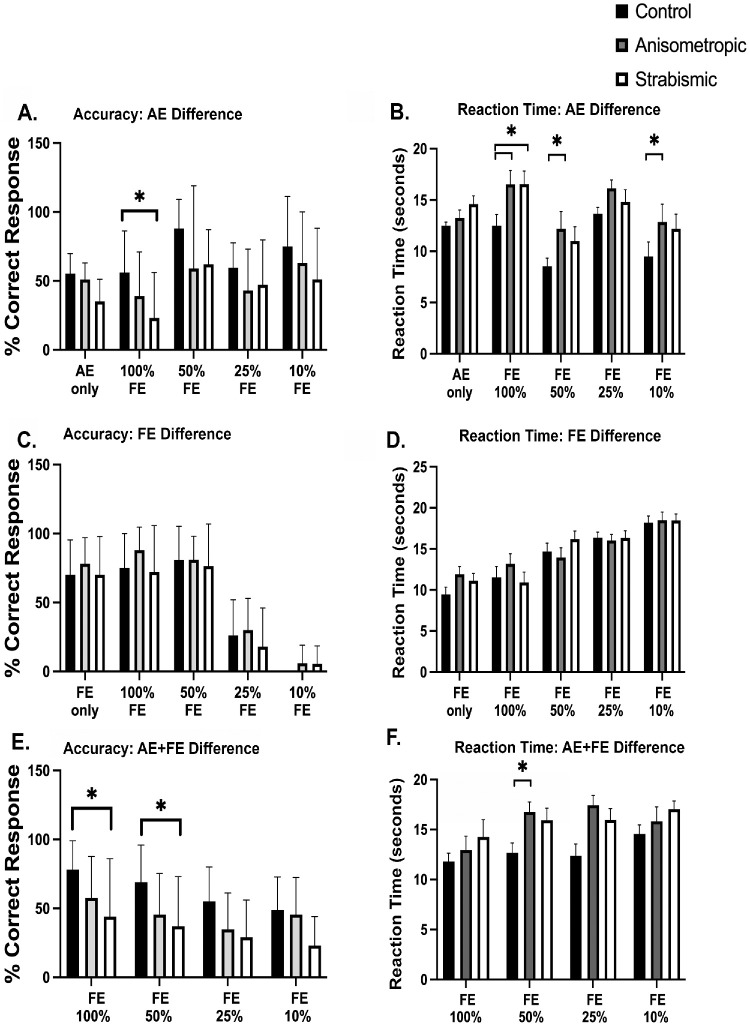
Accuracies and reaction times of controls, anisometropic and strabismic/mixed amblyopic participants in spot the difference tasks for AE difference (**A** and **B**), FE difference (**C** and **D**) and AE + FE difference (**E** and **F**). *Asterisk* indicates statistically significant difference between groups evaluated at *P* < 0.05 for post hoc multiple pairwise comparisons with Tukey correction.

Figure 6 summarizes the accuracies and reaction times of spot-the- difference tasks, when the difference is within the amblyopic eye region i.e. AE difference (Figs. 6A, 6B) and fellow eye region (i.e., FE difference; Figs. 6C, 6D) and when the difference was within AE and FE regions (i.e., AE + FE difference; Figs. 6E, 6F) in controls versus anisometropic and strabismic amblyopia participants. For AE difference, a significant main effect was found for accuracies between controls and amblyopic groups (*F*(2, 170) = 9.73, *P* < 0.0001). Significant differences were also observed within subjects for different viewing conditions for AE difference, (*F*(4, 170) = 5.88, *P* = 0.0002). No interactions were observed between the amblyopic and control groups and viewing conditions (*F*(8, 170) = 0.55, *P* = 0.84). Post-hoc comparisons revealed that participants with strabismic amblyopia had significantly lower accuracy in identifying AE difference compared to controls in 100% FE contrast trials (*P* = 0.01). Within control subjects, post hoc comparisons revealed improved accuracies at low FE contrast with significant differences between AE only versus FE 50% (*P* = 0.04) and FE 100% versus FE 50% (*P* = 0.04). Also, we observed improved accuracies in strabismic amblyopia participants at FE 50% compared to FE 100% (*P* = 0.01). Also, for AE difference, a significant main effect of reaction time was found between groups (*F*(2, 160) = 12.67, *P* < 0.0001), as well as within groups (*F*(4,160) = 8.33, *P* < 0.0001), with no interaction between groups and viewing conditions (*F*(8,160) = 0.64, *P* = 0.73). The reaction times were increased in anisometropic amblyopic participants compared to controls, with post-hoc comparisons revealing differences at FE 100% (*P* = 0.01), FE 50% (*P* = 0.01), and at FE 10% (*P* = 0.01). Increased reaction times were also observed in strabismic amblyopia subjects compared to controls at FE 100% (*P* = 0.02). Furthermore, within groups, improved reaction times were observed at 50% FE contrast compared to 100% FE contrast in strabismic amblyopia participants (*P* = 0.005).

For FE difference (Figs. 6C, 6D), no significant differences were observed in accuracy across groups (*F*(2,170) = 2.19, *P* = 0.11) with significant effects of viewing conditions (*F*(4,170) = 8.33, *P* < 0.00001) and no interaction observed across groups and viewing conditions (*F*(8,170) = 1.25). Similarly for reaction times, no significant differences were observed across groups (*F*(2,170) = 0.90, *P* = 0.40) with significant effects of viewing conditions (*F*(4,170) = 27.73, *P* < 0.00001) and no interaction observed across groups and viewing conditions (*F*(8,170) = 0.67). Post-hoc analysis revealed significant multiple pairwise comparisons between the various contrast levels for both amblyopia and control groups, showing that at lower FE contrasts (25% and 10%), participants had reduced accuracy and increased reaction times in both tasks, as expected.

Figures 6E and 6F plot the accuracies and reaction times for AE + FE difference trials across controls, anisometropic and strabismic amblyopia participants. We found a significant main effect of on accuracies (*F*(2,136) = 14.15, *P* < 0.0001) with post-hoc comparisons revealing that strabismic amblyopic participants were significantly less accurate than controls under 100% (*P* = 0.007) and 50% FE contrast (*P* = 0.01) trials. There was also an effect of viewing conditions on accuracies (*F*(3,136) = 4.46, *P* = 0.005), with post hoc comparisons showing reduced accuracies at 10% FE contrasts compared to FE 100% trials within control subjects (*P* = 0.03). No such differences were observed in amblyopic subjects, which is likely due to reduced accuracies at higher FE contrasts. For accuracies, no interactions were observed in different groups across viewing conditions (*F*(6,136) = 0.38, *P* = 0.88). For reaction times needed to identify AE + FE differences, a significant main effect was observed across groups (*F*(2,128) = 5.72, *P* = 0.004), as well as across viewing conditions (*F*(3,128) = 4.87, *P* = 0.003) with no interactions observed between groups across viewing conditions (*F*(6,128) = 0.79, *P* = 0.57). Post-hoc comparisons showed that anisometropic amblyopia had increased reaction times compared to controls at 50% FE contrast (*P* = 0.01). Thus the results suggest that amblyopic subjects experience difficulties in visual search and spot the difference tasks with greater deficits observed in strabismic participants particularly under high FE contrast conditions.

### Behavioral Performance Per Visual Function Deficits

Besides the type of amblyopia, visual acuity of the amblyopic eye, suppression and stereoacuity deficits could affect behavioral performance. Of note, for anisometropic versus strabismic/mixed amblyopic participants, there were no significant differences in visual acuity (logMAR) (Anisometropia = 0.30 ± 0.20, Strabismus/mixed = 0.27 ± 0.21, *P* = 0.93). Also, suppression levels were comparable between anisometropic (2.74 ± 0.54) and strabismic participants (2.68 ± 0.54) (*P* = 0.96). However, stereoacuity deficits (log arcsec) were greater in strabismic (2.84 ± 0.91) than anisometropic (2.20 ± 0.36) participants (*P* < 0.001). We examined how visual acuity in the amblyopic eye, suppression and stereoacuity deficits influenced accuracy and reaction times, using Spearman correlation analysis, as summarized in Table 2 for visual search and Table 3 for spot the difference tasks. For visual search AE targets (Table 2), strong correlations were seen indicating that participants with greater AE visual acuity deficits, greater stereoacuity deficits and higher suppression had less accurate identification with prolonged reaction times under AE-only and 100% FE contrast viewing conditions. These correlations persisted but weakened as FE contrast decreased to 50%, and FE 25% with no statistical differences observed at FE 10%. In contrast, for visual search FE targets, AE visual acuity, stereoacuity deficits and suppression levels did not exhibit significant relationship with accuracy or reaction times. For spot the difference AE targets (Table 3), moderate correlations were seen indicating that participants with greater AE visual acuity deficits, greater stereoacuity deficits and higher suppression had less accurate identification with prolonged reaction times under AE-only, 100%, 50%, and 25% FE contrast viewing conditions. No correlations between AE visual acuity, stereoacuity deficits and suppression were observed for accuracies or reaction times at 10% FE contrast. For FE differences, AE visual acuity deficit, stereoacuity deficits and suppression showed no relationship with accuracy or reaction times.

**Table 2.** Correlation Between Accuracies and Reaction Times During Visual Search Task and Visual Functions

Viewing Condition	Amblyopic Eye Visual Acuity	StereoAcuity	Suppression
AE only	***R* = −0.69, *P* = 0.0001**	***R* = −0.57, *P* = 0.0001**	***R* = −0.56, *P* = 0.0002**
	**[*R* = 0.65, *P* < 0.001]**	**[*R* = 0.59, *P* = 0.0001]**	**[*R* = 0.61, *P* = 0.0001]**
100% FE (AE target)	***R* = −0.65, *P* = 0.001**	***R* = −0.50, *P* = 0.0006**	***R* = −0.46, *P* = 0.002**
	**[*R* = 0.60, *P* = 0.0001]**	**[*R* = 0.70, *P* < 0.001]**	**[*R* = 0.42, *P* = 0.006]**
50% FE (AE target)	***R* = −0.34, *P* = 0.015**	***R* = −0.34, *P* = 0.015**	*R* = −0.11, *P* = 0.24
	**[*R* = 0.37, *P* = 0.012]**	**[*R* = 0.48, *P* = 0.001]**	[*R* = 0.18, *P* = 0.15]
25% FE (AE target)	***R* = −0.39, *P* = 0.008**	***R* = −0.51, *P* = 0.0006**	***R* = −0.47, *P* = 0.002**
	**[*R* = 0.37, *P* = 0.012]**	**[*R* = 0.50, *P* = 0.001]**	**[*R* = 0.51, *P* = 0.001]**
10% FE (AE target)	*R* = −0.13, *P* = 0.21	*R* = −0.18, *P* = 0.13	*R* = −0.02, *P* = 0.43
	[*R* = 0.27, *P* = 0.05]	[*R* = 0.27, *P* = 0.05]	[*R* = 0.12, *P* = 0.24]
FE only	*R* = 0.01, *P* = 0.47	*R* = −0.07, *P* = 0.33	*R* = 0.10, *P* = 0.27
	[*R* = 0.11, *P* = 0.24]	[*R* = 0.14, *P* = 0.19]	[*R* = 0.04, *P* = 0.41]
100% FE (FE target)	*R* = −0.002, *P* = 0.49	*R* = 0.06, *P* = 0.35	*R* = −0.07, *P* = 0.33
	[*R* = 0.18, *P* = 0.13]	[*R* = 0.16, *P* = 0.17]	[*R* = 0.18, *P* = 0.15]
50% FE (FE target)	*R* = 0.13, *P* = 0.20	*R* = 0.04, *P* = 0.40	*R* = 0.07, *P* = 0.33
	[*R* = −0.23, *P* = 0.08]	[*R* = 0.07, *P* = 0.32]	[*R* = 0.01, *P* = 0.47]
25% FE (FE target)	*R* = −0.14, *P* = 0.19	*R* = −0.18, *P* = 0.13	*R* = −0.28, *P* = 0.04
	[*R* = 0.06, *P* = 0.36]	[*R* = 0.19, *P* = 0.12]	[*R* = 0.20, *P* = 0.12]
10% FE (FE target)	*R* = 0.30, *P* = 0.03	*R* = 0.11, *P* = 0.24	*R* = 0.32, *P* = 0.02
	[*R* = −0.16, *P* = 0.17]	[*R* = −0.11, *P* = 0.26]	[*R* = −0.33, *P* = 0.02]

Values in brackets represent data for reaction times.

†values in bold indicate statistically significant correlations- with Bonferroni correction applied with significant p value defined as < 0.016.

**Table 3.** Between Accuracies and Reaction Times During Spot-the-Difference Tasks And Visual Functions

Viewing Condition	Amblyopic Eye Visual Acuity	StereoAcuity	Suppression
AE only	***R* = −0.52, *P* = 0.0004**	***R* = −0.35, *P* = 0.014**	*R* = −0.12, *P* = 0.23
	**[*R* = 0.60, *P* < 0.0001]**	**[*R* = 0.35, *P* = 0.014]**	[*R* = 0.15, *P* = 0.18]
100% FE (AE diff)	***R* = −0.41, *P* = 0.005**	***R* = −0.39, *P* = 0.007**	***R* = −0.42, *P* = 0.005**
	**[*R* = 0.46, *P* = 0.002]**	**[*R* = 0.54, *P* = 0.004]**	**[*R* = 0.38, *P* = 0.01]**
50% FE (AE diff)	***R* = −0.48, *P* = 0012**	***R* = −0.36, *P* = 0.012**	*R* = −0.27, *P* = 0.05
	**[*R* = 0.47, *P* = 0.002]**	**[*R* = 0.35, *P* = 0.014]**	**[*R* = 0.47, *P* = 0.002]**
25% FE (AE diff)	***R* = −0.38, *P* = 0.01**	***R* = −0.40, *P* = 0.006**	***R* = −0.40, *P* = 0.007**
	**[*R* = 0.37, *P* = 0.014]**	**[*R* = 0.35, *P* = 0.014]**	**[*R* = 0.42, *P* = 0.006]**
10% FE (AE diff)	*R* = −0.15, *P* = 0.18	*R* = −0.18, *P* = 0.13	***R* = −0.42, *P* = 0.006**
	[*R* = 0.23, *P* = 0.08]	[*R* = 0.29, *P* = 0.04]	**[*R* = 0.51, *P* = 0.001]**
FE only	*R* = −0.23, *P* = 0.08	*R* = −0.18, *P* = 0.14	*R* = −0.25, *P* = 0.07
	[*R* = 0.24, *P* = 0.07]	[*R* = 0.24, *P* = 0.08]	[*R* = 0.34, *P* = 0.02]
100% FE (FE diff)	*R* = −0.06, *P* = 0.35	*R* = −0.11, *P* = 0.24	*R* = −0.09, *P* = 0.28
	[*R* = 0.21, *P* = 0.10]	[*R* = 0.10, *P* = 0.28]	[*R* = 0.20, *P* = 0.12]
50% FE (FE diff)	*R* = 0.12, *P* = 0.23	*R* = 0.18, *P* = 0.14	*R* = 0.002, *P* = 0.49
	[*R* = 0.01, *P* = 0.46]	[*R* = 0.04, *P* = 0.38]	[*R* = 0.17, *P* = 0.16]
25% FE (FE target)	*R* = −0.10, *P* = 0.25	*R* = −0.06, *P* = 0.36	*R* = −0.17, *P* = 0.15
	[*R* = 0.06, *P* = 0.36]	[*R* = −0.009, *P* = 0.47]	[*R* = 0.08, *P* = 0.31]
10% FE (FE diff)	*R* = −0.001, *P* = 0.49	*R* = −0.15, *P* = 0.17	*R* = 0.10, *P* = 0.26
	[*R* = −0.02, *P* = 0.43]	[*R* = 0.18, *P* = 0.13]	[*R* = −0.14, *P* = 0.21]
100% FE (AE + FE diff)	***R* = −0.35, *P* = 0.014)**	***R* = −0.28, *P* = 0.04**	*R* = −0.23, *P* = 0.08
	[*R* = 0.24, *P* = 0.07]	[*R* = 0.26, *P* = 0.05]	[*R* = 0.25, *P* = 0.08]
50% FE (AE + FE diff)	*R* = −0.28, *P* = 0.047	***R* = −0.47, *P* = 0.001**	*R* = −0.23, *P* = 0.08
	**[*R* = 0.40, *P* = 0.008]**	**[*R* = 0.52, *P* = 0.0001]**	[*R* = 0.25, *P* = 0.07]
25% FE (AE + FE diff)	*R* = −0.03, *P* = 0.41	*R* = −0.20, *P* = 0.11	*R* = −0.20, *P* = 0.12
	[*R* = 0.001, *P* = 0.49]	[*R* = 0.21, *P* = 0.11]	[*R* = 0.30, *P* = 0.04]
10% FE (AE + FE diff)	*R* = −0.27, *P* = 0.05	*R* = −0.34, *P* = 0.02	*R* = −0.34, *P* = 0.018)
	[*R* = 0.17, *P* = 0.15]	[*R* = 0.34, *P* = 0.02]	[*R* = 0.30, *P* = 0.04]

Values in brackets represent data for reaction times. Values in bold indicate statistically significant correlations, with Bonferroni correction applied with significant *P* value defined as < 0.016.

For AE + FE differences, AE visual acuity and stereoacuity deficits correlated with reduced accuracies and increased reaction times particularly at high FE contrasts. Such differences were not observed at low FE contrasts. Interestingly, no correlations were observed between extent of suppression and ability to perform the AE + FE difference tasks.

## Discussion

The study aimed to evaluate the behavioral performance of amblyopic subjects and controls during visual search and spot-the-difference tasks, focusing on reaction times and accuracies. It examined performance under monocular viewing (with only the FE or AE viewing the stimuli) and dichoptic viewing, where the FE contrast was initially kept at 100% and then reduced. This approach aimed to quantify how the perception of the amblyopic eye changes with such modifications and how effectively the stimuli seen by both eyes are integrated to perform the task successfully. The findings revealed that visual search and the ability to spot differences were reduced in amblyopic subjects when viewing with the amblyopic eye, as expected. Interestingly, there was a marked deterioration in performance when the fellow eye stimuli were at 100% contrast. Reducing FE contrast led to some improvement in the ability to identify the object of interest, with greater enhancements observed in anisometropic than strabismic subjects. Additionally, reducing FE contrast not only enhanced the perception of the amblyopic eye but also facilitated the effective combination of information from both eyes, as evidenced by improved performance in FE + AE difference stimuli in spot-the-difference tasks.

Despite these improvements, amblyopic subjects continued to exhibit increased reaction times in identifying the object at lower FE contrasts. Furthermore, subjects with greater suppression, stereoacuity deficits, and visual acuity deficits in the amblyopic eye showed less improvement in performance at reduced FE contrasts. In our current study, we recruited 19 children and four adults, with the majority of children being older than nine years of age. Despite the relatively older cohort, we found that modulation of FE contrasts resulted in some improvement in perception, as reflected in enhanced dichoptic visual search performance. Earlier studies on dichoptic therapies, primarily based on adults, have shown improvements in visual acuity and stereoacuity beyond the traditional window of plasticity.^18^^,^^19^ Thus our data supports the possibility of improving the perception of amblyopic eye beyond the traditional critical periods.

Although it is often assumed that individuals with unilateral amblyopia can function normally in real-world conditions, research evidence suggests otherwise.^5^^,^^28^^,^^36^ These individuals exhibit binocular visual acuity deficits, lack of binocular summation, stereoacuity deficits and fixation instability.^15^^,^^37^^–^^40^ Consequently, individuals with amblyopia can experience functional vision difficulties even in binocular viewing conditions, including slower reading rates, difficulties with visual search^5^^,^^27^^–^^30^ and challenges in higher order visual processing skills such as visual attention. Conventionally, brain plasticity is thought to peak during a critical period in early childhood and decrease thereafter^41^ However, over the last three decades, numerous studies have shown that brain plasticity can be triggered beyond these critical periods through various new approaches designed to treat amblyopia beyond conventional methods such as the newer dichoptic therapies.^16^^–^^19^ The dichoptic treatments have demonstrated improvements in binocular visual acuity, visual acuity deficits of the amblyopic eye, and recovery of stereoacuity.^42^ Despite these advancements, the outcomes of dichoptic treatments remain mixed, highlighting the need for further research.^15^^,^^20^^,^^21^

In our previous research, we have used eye tracking to investigate how varying FE contrast during passive viewing of dichoptic videos influences eye movement patterns. Complementary masks were applied to the images such that each eye sees a different portion of the same image (i.e., parts of the image are visible only to the FE region, and other parts of the same image are visible only to the AE region). We observed that participants with greater suppression, visual acuity deficits, and stereoacuity deficits had less durations when their amblyopic eye was in the AE region. This eye movement pattern was observed during viewing of dichoptic videos when the FE contrast was at 100%. As the FE contrast was reduced, there was an improvement with increased durations of the AE in AE region increased.^43^ This suggests that reducing FE contrast can disrupt established suppression patterns.^44^^,^^45^ However, the impact of such stimuli modifications on the actual perception of the amblyopic eye remains unclear. Our current study investigates visual search behavior while viewing dichoptic scenes that approximate naturalistic viewing scenarios while quantifying the perception of the amblyopic eye by evaluating behavioral performance. Recent research suggests that anisometropic and strabismic amblyopia exhibit increased interocular suppression.^33^ During the experimental session, we tried using the nonius cross-alignment procedure to achieve alignment and fusion of the two eyes in subjects with strabismus. However, due to the transient visibility and inconsistent location of the nonius cross reported by several strabismic participants, combined with the fluctuating eye deviation in the dichoptic environment, we decided to maintain the original stimulus presentation without adjusting based on nonius cross measurements.^4^^,^^13^ We found that both anisometropic and strabismic amblyopic participants experienced difficulties in dichoptic visual search and spot-the-difference tasks, particularly at high FE contrasts. Interestingly, whereas amblyopic subjects showed greater difficulties in these tasks, the challenges were more pronounced at lower FE contrasts in the spot-the-difference task. This may be because the spot-the-difference task is more complex compared to the visual search task, as evidenced by reduced accuracies observed in both controls and amblyopic subjects. Additionally, we found that reducing FE contrasts facilitates effective binocular integration, as indicated by improved accuracies in trials with AE + FE differences.

We and others have previously demonstrated that suppression levels correlate with the extent of visual acuity deficits in the amblyopic eye.^13^^,^^14^ Therefore we also evaluated how the severity of amblyopic eye visual acuity deficits, stereoacuity deficits, and suppression affect accuracies and reaction times in visual search and spot-the-difference tasks, and how reducing FE contrast impacts these metrics. Our findings indicate that amblyopic eye visual acuity deficits, suppression, and stereoacuity deficits correlate with AE target detection in both visual search and spot-the-difference tasks, particularly at high FE contrasts. For visual search tasks, no correlation was observed between accuracies and reaction times at 10% FE contrast and suppression levels. However, in the spot-the-difference task, subjects with greater suppression experienced persistent difficulties with reduced accuracies and prolonged reaction times even at 10% FE contrast. The observed higher reaction time in participants with amblyopia could possibly be due to their more conservative response criteria particularly in a more complex task such as spot-the- difference task. Alternatively, it could be attributed to inefficient visual search, where it takes them longer to reach the region of the target of interest.^28^^,^^30^ Additionally, once they reach the target, it may take them longer to process the target information. An eye tracker analysis could indeed provide valuable insights by showing fixations around the target some time before the response. This would help in understanding whether the delay is due to prolonged visual search or extended processing time at the target location. Further research incorporating eye tracking data could help clarify these mechanisms and improve our understanding of the visual processing challenges faced by individuals with amblyopia.

Finally, we also found that reducing the FE contrast in trials requiring effective binocular integration of FE + AE stimuli (i.e., AE+FE difference trials) led to reduced accuracies and prolonged reaction times at high FE contrasts for those with greater amblyopic eye visual acuity deficits and stereoacuity deficits. At 10% FE contrast, individuals with greater stereoacuity deficits continued to exhibit reduced accuracies despite the reduced FE contrast. This suggests that for more complex tasks, stereoacuity deficits impede performance in a way that cannot be overcome by reducing FE contrast alone. Thus this study addresses gaps in our understanding of how modifying the FE stimuli affects the perception of the amblyopic eye in various complex scenes. We find that some individuals were able to successfully overcome suppression at higher FE contrasts whereas others were not able to overcome suppression even at 10% FE contrast. We also observed that FE 25% and FE 10% as the contrast was so low it was difficult to visualize and spot the targets during dichoptic visual search or spot the picture differences in controls. This is important as the current FDA approved amblyopia therapy which utilizes dichoptic masking with modulation of FE contrast is administered at a fixed FE contrast of 15%. Our study provides novel data that perhaps modulating the FE contrast that low really is not truly binocular treatment as the FE contrast at 25% and 10% reduces effective binocular integration of stimuli presented to AE and FE simultaneously. Further our study highlights the individual variability in behavioral performance in response to FE stimuli modification. Thus dichoptic visual search and spot the difference tasks can be a useful tool to assess perception in dichoptic viewing, which can be useful to personalize stimuli for treatment and investigate treatment outcomes. Understanding the factors that mediate visual search performance can elucidate how baseline patient characteristics influence stimulus perception and explain the factors contributing to variability in treatment responses. Furthermore, this approach can identify optimal stimuli that facilitate binocular integration and personalize treatment, thereby enhancing the effectiveness of training programs.

Despite the valuable insights gained from this study, several limitations should be noted. Previous research has shown that individuals with amblyopia exhibit higher-order perceptual deficits, suggesting abnormal processing in both the ventral “what” and dorsal “action” pathways, as well as difficulties with tasks involving higher-order attentional components.^46^^–^^49^ However, we did not examine how these deficits impact the ability to perform visual searches. Functional vision, which refers to the use of vision for daily living activities, can be measured by tasks that closely resemble everyday activities (e.g., searching for a pair of scissors). Visual search tasks have been used to assess functional vision and attention in children and adults with amblyopia under fellow eye, amblyopic eye, and binocular viewing conditions.^27^^–^^30^ A study on adults with amblyopia using non-natural targets (Gabor patches) found that subjects with amblyopia took longer search times only for conjunction visual searches, not for feature search tasks. This indicates that tasks requiring feature binding and higher cognitive inputs can be impaired in amblyopia.^27^^–^^30^ Performing visual searches with real world images in naturalistic viewing conditions can introduce bias because of the varying difficulty of the objects of interest. However, this approach more accurately reflects real-world functional vision tasks. To reduce bias, we collected data from age-matched controls to compare the behavioral performance of amblyopic participants using the same stimuli. Another limitation is that stereoacuity was assessed using the Titmus Fly Test, which, although commonly used, may yield false results because of the influence of monocular and non-stereoscopic binocular cues. Besides these, the relatively small sample size restricts the generalizability of our findings. This smaller cohort size precluded detailed analyses of how suppression, stereoacuity, and visual acuity deficits within each clinical type of amblyopia influence behavioral performance. Additionally, the study was observational and conducted at a single time point. Future studies should involve larger cohorts and longitudinal designs to observe changes over time.

In summary, despite its limitations, this study helps bridge gaps in our understanding of how modifying FE stimuli impacts the perception of the amblyopic eye in various complex scenarios. By examining the effects of visual acuity deficits, suppression, and stereoacuity deficits, we gain valuable insights into the challenges faced by individuals with amblyopia and how these factors influence their performance in visual tasks. In conclusion, although dichoptic treatments show promise, the mixed results highlight the need for ongoing research. Quantifying the perception of the amblyopic eye in dichoptic environments using tools like dichoptic visual search could help tailor therapies that typically involve passive dichoptic videos. Also, advanced methods like dichoptic visual search could provide deeper insights into neural mechanisms and contribute to the development of more optimal amblyopia therapies.
